# Subcutaneous and lung metastases from chondrosarcoma of the thumb

**DOI:** 10.1259/bjrcr.20150129

**Published:** 2015-08-17

**Authors:** R K Lalam, V N Cassar-Pullicino, N Kumar, W P Cool, G L Cribb, D C Mangham

**Affiliations:** ^1^ Department of Diagnostic Imaging, Robert Jones and Agnes Hunt Hospital, Oswestry, UK; ^2^ Department of Orthopaedic Oncology, Robert Jones and Agnes Hunt Orthopaedic Hospital, Oswestry, UK; ^3^ Department of Musculoskeletal Pathology, Robert Jones and Agnes Hunt Orthopaedic Hospital, Oswestry, UK

## Abstract

We present a rare case of metacarpal chondrosarcoma with cutaneous metastases in the ipsilateral upper arm. Chondrosarcomas of the small bones of the hand rarely metastasise unlike chondrosarcomas elsewhere in the body. Excision/ray amputation rather than curettage may be preferable in the treatment of high-grade chondrosarcomas in the hand.

## Summary

Chondrosarcoma of the hand is a rare malignant tumour of the bone, which usually is slow growing and has an extremely low metastatic rate. We report a rare case of subcutaneous and pulmonary metastases from a metacarpal chondrosarcoma 10 months after ray amputation but without local recurrence.

## Introduction

Chondrosarcoma is one of the most frequent primary bone malignancies in adults aged over 40 years (incidence male : female 2 : 1).^1^ The most common sites of involvement are the long tubular bones (50%). In contrast, while enchondromata are common, chondrosarcoma of the small bones of the hand is extremely rare. Chondrosarcoma can metastasize to the lung, followed by the skin, soft tissues and, rarely, to other bones.^2^ Here we illustrate a rare case of a grade II chondrosarcoma of the thumb metacarpal with subcutaneous and pulmonary metastases. There are only three previously reported cases of chondrosarcoma of the small bones of the hand with cutaneous metastases in the literature. Our case demonstrates certain differences from the previous reports.

## Case report

**Figure 1.**
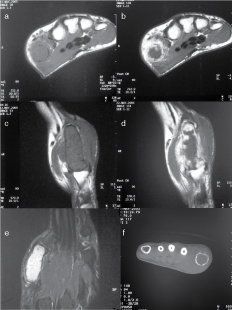
*T*_1_ weighted pre-contrast axial (a) sagittal (c) and corresponding post-contrast MRI axial (b) and sagittal (d) from May 2003 show a low-signal lesion with extensive destruction and expansion of the bone; following contrast medium, the lesions have an inhomogeneous, multilobulated, peripheral enhancement but also areas of non-enhancement predominate centrally, consistent with the cartilaginous nature of the lesion. This is associated with a soft-tissue mass and an extraosseous component, primarily on the dorsal aspect. Coronal short tau inversion-recovery image (e) from May 2003 shows a lesion of high signal with a thin and irregular septae, which involves the entire shaft of the first metacarpal bone. Axial CT image from May 2003 (f) shows a lytic lesion involving the first metacarpal of the left hand with significant expansion and thinning of the cortex circumferentially; there is a breach in the cortex and some new bone formation on the dorsal aspect. Some soft-tissue swelling on the dorsal aspect of the metacarpal shaft is also seen.

We describe a case of a 40-year-old female nurse who presented in May 2003 with an 18-month history of pain in the left thumb. Clinical examination showed soft-tissue swelling around the left thumb metacarpal. Radiographs showed an expansile lesion occupying most of the metacarpal with periosteal reaction and cortical destruction. MR scan (Figure 1) showed extensive destruction with expansion of the shaft of the first metacarpal of the left hand, associated with a soft-tissue mass and an extraosseous component, primarily on the dorsal aspect. On *T*_1_ weighted images, the lesion was of low signal; on fluid-sensitive sequences, the lesion showed high signal with a thin and irregular septae. Following contrast medium administration, inhomogeneous, multilobulated, peripheral enhancement of the lesion was noted but also areas of non-enhancement predominated centrally, consistent with a cartilaginous lesion.

**Figure 2.**
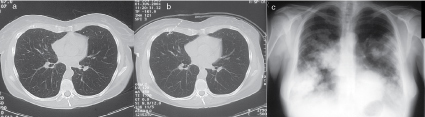
Chest CT image from June 2003 (a) shows no pathological findings in the anterior part of the middle lobe of the right lung. Chest CT scan from June 2004 (b) of the same location, as in Figure 2a, shows a subpleural, spiculated nodule (arrow) in the anterior part of the right lung middle lobe. The chest radiograph from August 2006 (c) shows multiple lung metastases from chondrosarcoma.

The CT scan (Figure 1f) demonstrated a lytic lesion involving virtually the entire shaft of the first metacarpal of the left hand and reaching the subchondral bone plate at both ends. The lesion demonstrated a significant expansion of the bone with small areas of punctuate calcification, significant thinning of the cortex circumferentially and a breach in the cortex with some new bone formation on the dorsal aspect. Some soft-tissue swelling was also seen. There was no evidence of pulmonary metastases on the CT scan (Figure 2a) at initial diagnosis.

**Figure 3.**
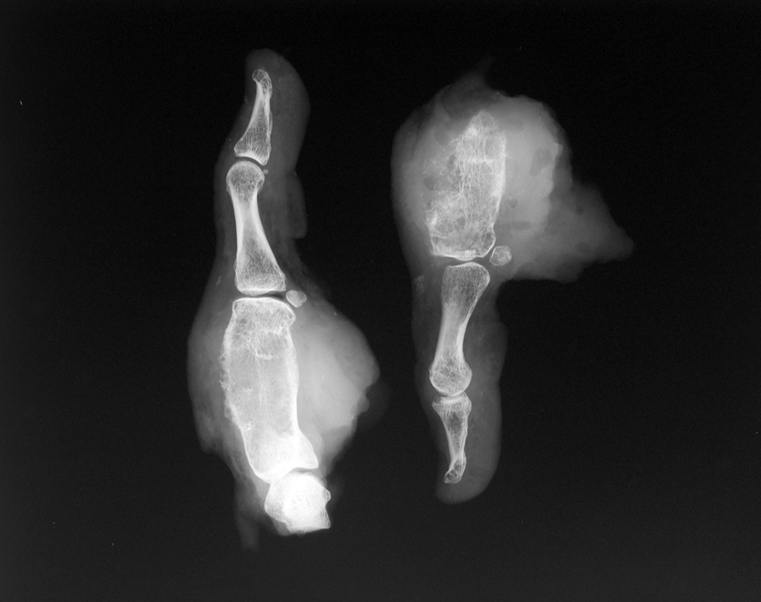
Anteroposterior radiographs of the specimen after ray amputation that show an expansile lytic lesion occupying most of the metacarpal and reaching the subchondral bone plate at both ends without joint involvement. Periosteal reaction and cortical destruction is noted.

An open biopsy was performed in June 2003, the histology of which revealed a grade II chondrosarcoma with evidence of spread beyond the cortex into the periosteum. Vascular invasion was not identified. 1 month later, under general anaesthesia, a first ray amputation was performed with disarticulation of the thumb at the trapezioscaphoid joint. A tourniquet was used at the time of the surgery to exsanguinate the limb. Macroscopic examination revealed an expansion of the metacarpal bone due to a chondroid tumour measuring 35 × 25 mm, which eroded through the cortex but did not cross the metacarpophalangeal joint. Microscopic examination of the sections (Figure 3) confirmed a grade II chondrosarcoma, which showed an extensive permeative growth beyond the cortical bone. There was no de-differentiation and the tumour was contained within the periosteum. The lesion was completely excised, although the closest margin was 1 mm on the dorsal aspect.

**Figure 4:**
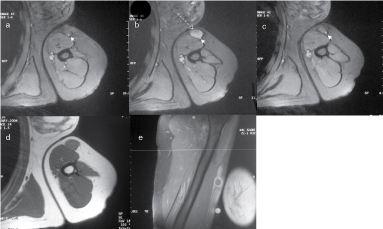
Gradient echo axial MRI from May 2004, above (a), at the level of (b) and below (c) the subcutaneous lesion in the proximal upper arm. The lesion (arrow) is well defined, demonstrates high signal and is closely related to the cephalic vein (arrow heads). *T*_1_ weighted axial spin-echo pre-contrast (d) and post-contrast *T*_1_ fat-saturated coronal (e) MRI from 2004 reveal a well-defined subcutaneous nodule that is of intermediate low signal on *T*_1_ weighted pre-contrast images and after intravenous gadolinium administration shows peripheral rim enhancement.

In April 2004, the patient noticed a superficial, firm swelling in the medial aspect of the left upper arm, which was not particularly painful. There was no clinical or radiographic evidence of local recurrence in the hand. An MRI scan demonstrated a subcutaneous lesion (Figure 4) on the medial aspect above the elbow with signal characteristics similar to the previous chondrosarcoma in the ipsilateral hand. The mass was closely related and in the line of the cephalic vein; it showed high signal on the *T*_2_ weighted images, intermediate signal on the *T*_1_ weighted images and peripheral enhancement after intravenous gadolinium administration. Subsequently, an excisional biopsy of the left upper arm lesion was performed. Macroscopically, the soft-tissue mass showed encapsulated grey myxoid tissue, which was centrally cystic and partially haemorrhagic. This was microscopically confirmed to be a grade II chondrosarcoma, most likely representing a metastasis from the previous thumb chondrosarcoma. There was no lymphoid tissue seen that could be associated with the subcutaneous lesion to suggest lymph node metastases. The closest margin was less than 1 mm. At the same time, a biopsy of the hypertrophic scar at the site of ray amputation was performed, which did not reveal any evidence of neoplasia on histological examination. Owing to the close margins of this subcutaneous lump on excision, a wider excision was performed a month later under general anaesthesia.

In June 2004, a CT scan of the chest was performed and a solitary tiny nodule in the right middle lobe (Figure 2b), which was suspicious for lung metastasis, was seen. There was no mediastinal or hilar lymphadenopathy seen. The lesion continued to increase in size and the patient had a pulmonary lobectomy in November 2004. Within 2 months, however, she developed further lung metastases. In January 2005, she received palliative chemotherapy, but even after three cycles, the pulmonary lesions continued to increase in size and number (Figure 2c) and it was decided to discontinue further treatment. The patient died subsequently from the disease.

## Discussion

Chondrosarcoma is the second most common primary malignant bone tumour. The long bones are the most common site of this tumour. Benign cartilaginous tumours are common in the hand but chondrosarcoma is rare and represents less than 2% of all cases, as demonstrated in a large Mayo Clinic study.^2,3^ Histological grading of cartilaginous tumours of the hand is difficult and often variable. Sometimes, it can be difficult to distinguish histologically a well-differentiated chondrosarcoma from an enchondroma; thus, a benign lesion is sometimes referred to as “atypical enchondroma” or “cartilaginous lesion of unknown malignant potential” to distinguish it from a lesion that is frankly malignant.^4–6^ Chondrosarcomas are commonly classified histologically into low (grade I), intermediate (grade II) and high-grade malignant (grade III). Their malignant potential (predictor of local recurrence and metastases) is based on Evans’ nuclear grading.^3^ As many studies have already demonstrated, the survival is related to tumour grade.^1,4–6^ Sankerin et al^4^ reported a 10-year survival rate of 89% in low-grade, 71% in intermediate-grade and 38% in high-grade tumour, and similar data were described by Lee et al^1^ and Fiorenza et al.^5^ However, in the study by Fiorenza et al,^5^ no distinction was made between de-differentiated chondrosarcoma and grade III chondrosarcoma, which are distinct tumours, and a large proportion of their patients had wide resection of tumours, unlike other studies, which accounts for their lower recurrence rates. It has already been well demonstrated that the nature of surgical treatment is a very important prognostic indicator, with a wider resection demonstrating better survival and lower recurrence rate than curettage.^1,4^ All these studies deal with chondrosarcomas in general and not specifically with cartilaginous lesions of the hand. Chondrosarcomas of the hand are generally thought to be of low-malignant potential and are usually dealt with conservatively. Given the grade of the tumour in our case on the initial open biopsy, a decision was made to perform an amputation rather than a curettage.

Chondrosarcomas of the hand usually display benign biological behaviour even when they demonstrate cytological and imaging features suggestive of malignancy. However, some cases, as reported in the literature, showed local recurrence and could metastasize.^7^ Little is known about the biological behaviour of phalangeal chondrosarcoma and controversial data are reported in the literature.^8–13^ Bovèe et al^8^ have previously reported that Evans’ grading could not be applied to chondrosarcoma of the hand in predicting disease-free survival, because it appears to display a different behaviour from chondrosarcomas located elsewhere in the body. In addition, the radiological and histological findings by themselves are not always reliable in distinguishing phalangeal chondrosarcoma from enchondroma. Thus, a combination of clinical, histological and imaging features are important to differentiate low-grade chondrosarcomas from enchondromas of the hand.

Similar to chondrosarcomas located elsewhere in the body, the incidence of local recurrences in chondrosarcoma of the hand relates to surgical treatment.^11,14^ In a large study from Mayo Clinic, all patients treated with curettage had a higher rate of local recurrence than those treated with ray resection or amputation.^2^ Bovèe et al^8^ reported a recurrence rate of 36% and it was likely owing to the low proportion of radical surgical treatment performed in their study.^8^ There is a relative lack of vascularity in cartilage tumour matrix and the neoplastic cells appear to depend on diffusion for nourishment. This probably allows cartilaginous lesions to be easily transplantable lesions, giving rise to recurrences.^15^ Chondrosarcomas metastasize usually by the haematogeneous route and rarely by the lymphatic route. Metastases occur when the tumour erodes the vein wall and forms intravenous plugs; neoplastic thrombi then develop within the venous tree and migrate to the lung. Thus, metastases are nearly always pulmonary and rarely to other sites, including the skeleton or the skin, in the absence of pulmonary metastases.^16^ Metastases can, however, occur after a significant period of time ranging from a few months to many years after the onset of symptoms.^7,10,11^

Most previously reported chondrosarcomas metastatic to the skin originated in the bones of the extremities. These metastases can be either single or multiple with a slight predilection for the head and neck region. A review of the published papers of skeletal chondrosarcomas metastatic to the skin yielded a total of nine cases.^11,17–24^ There were, however, only three previous case reports of cutaneous metastases from a chondrosarcoma of the hand.^11,17,18^ The earliest report by Cruickshank^17^ involved a middle phalangeal chondrosarcoma with cutaneous and pulmonary metastasis. This patient developed multiple nodules in the skin of the face, neck and trunk. Karabela-Bouropoulou et al^11^ have reported a case of thumb chondrosarcoma with pulmonary and subcutaneous metastasis that developed 6 years after the initial diagnosis. Although many studies^8,9^ have underlined the low risk of metastases in chondrosarcomas of the hand compared with those located elsewhere, the previous case of chondrosarcoma of the hand with skin and lung metastases reported by Karabela-Bouropoulou et al^11^ and the present report demonstrated a very aggressive behaviour and both patients died soon after the appearance of cutaneous metastases.

As described above, a satisfactory wide resection of hand chondrosarcoma is usually associated with a very good long-term prognosis.^4–6^ Our patient noticed the lump in her arm 11 months after resection of the primary tumour and lung metastasis was discovered 1 month later. Cutaneous metastases from chondrosarcomas usually occur in the trunk, neck or face. There was only one previously reported case of solitary subcutaneous metastasis from a chondrosarcoma of the hand localized to the ipsilateral upper limb.^18^ In this case, the author suggested strong clinical evidence in keeping with mechanical tumour transplantation at the site of Bier block access for anaesthesia, which later developed into cutaneous metastasis. The Bier’s block is an intravenous regional analgesia and is best used for brief minor surgery of the hand and the forearm. An intravenous cannula or butterfly needle is inserted in a distal vein in the limb scheduled for surgery. A tourniquet is then applied to the upper arm. In our case, we could not identify any reason for direct tumour transplantation and our patient had a general anaesthetic for biopsy and resection rather than regional anaesthesia. However, in our patient, a blood pressure cuff was placed in the ipsilateral limb above the elbow, but the limb was never exsanguinated. The subcutaneous metastasis developed just distal to the site of the cuff. In addition, the skin metastasis was related closely to the cephalic vein, which drains the radial part of the dorsal network of veins of the hand, in particular the thumb and the index finger. Although unproven, it is plausible that there may have been venous dispersal of the tumour at surgery or during the open biopsy through the cephalic vein that lodged itself in the subcutaneous tissue just distal to the blood pressure cuff owing to the occlusive pressure on the veins by the cuff. Of course, the metastatic seedling could also have occurred prior to surgery owing to venous invasion by the tumour itself.

## Conclusions

We present a rare case of cutaneous metastasis in the ipsilateral upper limb from a metacarpal chondrosarcoma and suggest a plausible cause for its occurrence. The potential for metastases of chondrosarcomas of the hand is much lower than for chondrosarcomas of other parts of the body. Additional and more specific studies are necessary to clarify the behaviour of chondrosarcomas of the hand related to different histological subtypes. However, the authors believe that for grade II and III chondrosarcomas, wide excision rather than conservative treatment should be preferred in order to avoid local recurrence or metastasis, and adequate precautions to prevent transplantation of the tumour cells to other sites during surgical procedures are necessary. Since metastases can develop long after the initial diagnosis, these tumours should be followed up very closely.

## Learning points

1. This is a very rare and unusual case of metacarpal chondrosarcoma with subcutaneous and pulmo-nary metastases.
2. Hand chondrosarcomas are seldom metastatic in nature. Death is virtually unknown. Pulmonary metastases from hand chondrosarcomas are rare but not unknown.
3. For higher grade chondrosarcomas of the hand, excision/ray amputation is probably a better treatment than curettage.
4. This article also shows a rare occurrence of subcutaneous metastases from small bone chondrosarcoma, which is relatively unknown.
5. It is plausible that inflatable cuffs, such as blood pressure monitoring devices and tourniquets, may have a role in determining where metastatic deposits may occur.
